# Insulin concentrations used in in vitro embryo production systems: a pilot study on insulin stability with an emphasis on concentrations measured in vivo

**DOI:** 10.1186/s13028-016-0249-9

**Published:** 2016-10-20

**Authors:** Denise Laskowski, Ylva Sjunnesson, Hans Gustafsson, Patrice Humblot, Göran Andersson, Renée Båge

**Affiliations:** 1 Division of Reproduction, Department of Clinical Sciences, Swedish University of Agricultural Sciences, Uppsala, Sweden; 2 Department of Animal Breeding and Genetics, Section of Molecular Animal Genetics, Swedish University of Agricultural Sciences, Uppsala, Sweden

**Keywords:** In vitro model, Blastocyst, Bovine, Embryo development, Oocyte maturation, Insulin

## Abstract

**Background:**

Insulin has been used as a stimulatory factor for in vitro cell culture since many years. Even for routine in vitro embryo production (IVP), insulin is added to the media during different steps. There is a strong difference in concentrations used in vitro compared to what is measured in vivo in follicular fluid or serum. We performed a pilot study on insulin stability to explain possible reasons for that variation.

**Results:**

We measured insulin concentrations before and after bovine oocyte maturation in an experiment by using a quantitative ELISA (Mercodia bovine insulin ELISA immunoassay) and found that concentrations were stable up to 22 h of incubation. We compared our results with eleven in vivo studies measuring insulin in either serum or follicular fluid and nine IVP-protocols using insulin. In all studies, in vitro concentrations were much higher compared with those found physiologically in vivo. Limited knowledge is available concerning the different activity and stability of insulin in vitro versus in vivo.

**Conclusions:**

The concentrations of insulin used in vitro are quite high in comparison to physiological concentrations found in serum or follicular fluid. One explanation may be a different stability or activity of insulin in vitro even if we could measure stable concentrations of insulin in our pilot study. More precise dose–effect studies have to be performed to draw clear conclusions about the consequences of the use of such high doses as they might have negative consequences for the developing embryo. Insulin has direct effects on the regulation of the metabolism and could even influence the epigenetic programming of the metabolism with unknown consequences for the offspring later in life.

## Findings

In vitro production (IVP) of embryos is currently performed according to standardized protocols for many species and for both commercial and scientific reasons. Oocytes are matured, fertilized and presumed zygotes are cultured in the laboratory until blastocyst stage. Media used in the laboratory usually aim to simulate the natural milieu as closely as possible, e.g. by using synthetic oviductal fluid (SOF) with or without serum for embryo culture. Different protocols use insulin as a stimulatory factor (well known from cell culture as reported in 1976 [1]) to improve embryo development rates.

We performed a pilot study with the aim of further investigating insulin stability in embryo production systems as there is, according to our knowledge, no clear evidence for the choice of the deviating concentrations in vitro.

The media used in our lab contains cysteine in the TCM199 (M2154, Sigma-Aldrich, Stockholm, Sweden).

We added insulin to the in vitro maturation medium consisting of bicarbonate-buffered TCM199 (M2154) supplemented with 0.68 mM l-glutamine (G8540), 0.5 µg/ml follicle-stimulating hormone (FSH) and 0.1 µg/ml luteinizing hormone (LH) (Stimufol; PARTNAR Animal Health, Port Huron, Canada), 50 µg/ml gentamicin and 0.4 % w/v bovine serum albumin (BSA). Groups of 30–45 bovine cumulus oocyte complexes (COCs) were matured in a separate well containing 500 µl medium supplemented either with 0 (INS0), 0.1 µg/ml (INS0.1) or 10 µg/ml (INS10) bovine insulin (I5500). All COCs were incubated for 22 h (24 h after the end of aspiration) at 38.5 °C under a 5 % O_2_, 5 % CO_2_ atmosphere. Insulin concentrations were analysed in three different conditions: a) before maturation when the medium itself had been incubated around 2 h for equilibration of temperature and atmosphere and before any COCs were added (BM); b) after maturation with co incubation of COCs for 22 h (AM) and c) after maturation “empty” (AME), a control containing only media without any COCs incubated for 22 h. The Mercodia bovine insulin ELISA Immunoassay for quantitative determination of bovine insulin in serum or plasma specially optimized for bovine samples, was used (Tables 1, 2). The two different insulin concentrations were used to permit the evaluation of a more extreme dose compared to a dose closer to physiological concentrations. The study size did not allow any advanced statistics, only numerical result assessment. Stable concentrations of insulin were found as the added concentrations (0.1 and 10 µg/ml) related well to the measured concentrations after 22 h of incubation. Summarizing, the results of the pilot study do not support the theory that insulin degradation is a reason for using higher insulin concentrations in in vitro studies. However, no statements about the biological activity can be made based on this quantitative ELISA.

**Table 1:** Insulin concentration in maturation media measured immediately or after 22 h of incubation (INS10 = 10 µg/ml added insulin)

Sample identity	Treatment	Bovine insulin concentration (μg/ml)
1709	Before maturation	5
	After maturation without oocytes added	4.1
	After maturation with co incubation of oocytes	3
2711	Before maturation	4.6
	After maturation with co incubation of oocytes	5
2611	After maturation with co incubation of oocytes	3.1

**Table 2:** Insulin concentration in maturation media measured immediately or after 22 h of incubation (INS0.1 = 0.1 µg/ml added insulin)

Sample identity	Treatment	Bovine insulin concentration (μg/ml)
1709	Before maturation	0.24
	After maturation without oocytes added	0.19
	After maturation without oocytes added	0.16
2611	After maturation with co incubation of oocytes	0.13
2711	Before maturation	0.15
	After maturation without oocytes added	0.11

To put our study in the context of recent research, a comparison to recent studies using insulin was made, summarized in Tables 3 and 4.

**Table 3:** Insulin concentrations used for in vitro experiments based on published literature

Insulin during IVM (µg/ml)	Insulin during IVC (µg/ml)	References	Main finding
10	10	[2]	No effect on BC rate
5 (*)	–	[3]	No effect on BC rate nor cell number
1	–	[4]	No effect on BC rate, cell number elevated
0.1/0.5/1/10	–	[5]	0.5–10 μg/ml stimulated development until morula
5 (7, 12 or 22 h)	–	[6]	Difference as different co-factor
–	5 (¤)	[7]	BC rates similar to those obtained by serum addition
–	5 (¤)	[8]	Insulin alone no beneficial effect on BC rates
–	0.01 and 10 (^®^)	[9]	Insulin no effect on BC rate nor cell number but negative effect on apoptosis
–	ED50 0.1 for granulosa cell mitogenesis ED50 0.01 for estradiol production ED50 for 0.1 for progesterone production ED50 0.2 for androgen production	[11]	ED50 depends on different aspect of insulin action

*IVM* in vitro maturation, *IVC* in vitro culture, * follicular culture, *BC* blastocyst, (¤) *ITS* insulin/transferrin/sodium selenite added to serum free SOF (synthetic oviductal fluid, ^®^) reported units 1.8 pM/1.8 nM, insulin = 180.16 mg/mol, ED50 = median effective dose

**Table 4:** Insulin concentrations measured in follicular fluid and serum based on published literature

Insulin in plasma (ng/ml)	Insulin in follicular fluid (ng/ml)	Method/kit	References
0.21–0.48	–	A specific double antibody RIA	[13]
0.32–0.40	–	125I-labeled insulin Double-antibody RIA	[14]
0.42–1.88	–	125I-labeled insulin Double-antibody RIA	[15]
0.349–0.712 Luteal phase = 0.349; Follicular phase = 0.417; Peak = 0.712; Mean = 0.5–0.69	0.127–0.282 Subordinate = 0.12–0.17; Preovulatory = 0.3–0.72	RIA Coat-A-Count kit (DPC, Los Angeles, CA, USA) validated for cattle by [16]	[17]
–	0.5–10	–	[11]
0.21–0.34	–	ELISA, validated for cattle (Immuno-Biological Laboratories, Hamburg, Germany)	[18]
–	0.38–0.42	By EIA not specified	[19]
–	0.59 (acyclic) 1.01 (cyclic)	RIA (RIAK-1 kit, BRIT, Navi Mumbai, India)	[20]
–	0.27 (cyst) 1.15 (normal)	RIA (RIAK-1 kit, BRIT, Navi Mumbai, India)	[21]
0.24 (heifer) 0.51 (cow)	–	Insulin auto DELFIA (PerkinElmer Life and Analytical Science, Turku, Finland)	[22]
0.4–0.6	–	EIA	[23]

*RIA* radio immunoassay, *ELISA* enzyme-linked immunosorbent assay, *EIA* enzyme immunoassay, *DELFIA* dissociation-enhanced lanthanide fluoro immunoassay

In the referred studies, insulin is added during the 22 h of in vitro oocyte maturation [2–6], or added to the culture medium during early embryo development [7–9]. Most concentrations used vary between 5 and 10 µg/ml (Table 3). Different effects have been reported, and both beneficial and no significant effects on development have been shown. Even a change in phenotype, in form of increased cell numbers in the blastocyst, has been reported after insulin exposure during oocyte maturation [4, 10]. This could be a sign of accelerated growth of the embryos deriving from these conditions with potentially harmful consequences.

Several studies have been published measuring insulin concentrations in serum and some measuring insulin in follicular fluid, which generally contains hormones and metabolites in concentrations close to those found in serum [11]. The use of different analyzing methods and units (IU/ml; µg/ml; µM/ml) makes comparisons complicated. Most of these studies report concentrations between 0.1 and 1 ng/ml in the bovine (Table 4). The values indicated in the tables are all transformed to the same units; µg/ml in Table 3, respective ng/ml in Table 4, based on the molecular weight of insulin 180.16 mg/mol; 1 mg insulin equals 25.7 or 1 IU = 0.03891 mg.

In the in vivo situation, the developing follicle and maturing oocyte will have a longer exposure to metabolic substances such as insulin. The shortened exposure time might be one factor that could explain that in vitro systems in research often are performed with higher doses than those found physiologically. Another explanation is that in general, in vitro systems have to be pushed with higher concentrations in order to eliminate noise of other influences during the investigated period.

Moreover, the use of supra-physiological doses could be justified by the fact that insulin has been reported to be quite unstable in vitro, due to media containing cysteine [12]. That study demonstrated that a minimum concentration of 1 µg/ml was necessary to stimulate cells in culture resulting in a maximal stimulation 1 h post exposure. Insulin action thus seems to be immediate on the cell. On the other hand, Spicer and Echternkamp [11] report an effective dose 50 (ED50) between 0.01 and 0.2 µg/ml (depending on the effect, as e.g. granulosa cell mitogenesis or estradiol production) in ovarian cell culture, which is closer to the physiological concentrations. These authors used Dulbecco’s Modified Eagle Medium and Ham’s F12 (DMEM/F-12) medium which also contains cysteine.

Knowing that conditions occurring with hyperinsulinemia in vivo (metabolic syndrome, obesity or diabetes) impair fertility, the use of insulin in non-physiological doses should be used cautiously.

Insulin is used in IVP as its stimulatory effects on growth and proliferation have been seen as beneficial for the embryo development while possible negative consequences for the epigenetic regulation and metabolic programming have been largely ignored. Research models should aim to work with concentrations as close as possible to the physiological conditions. Our pilot study does not support insulin degradation as a cause for using 100–1000-fold higher insulin concentrations in in vitro systems than can be found in vivo. Even if no statement about insulin activity can be made, the molecular structure of insulin seems to remain stable—as detectable by ELISA—even in media containing cysteine as in our maturation media based on TCM199. The gap between added and measurable concentrations in the INS10 groups (Table 1) could be explained by the fact that the samples needed to be diluted for the ELISA to remain in the measurement window and this could lead to less accuracy especially as the sample size is low. As the concentrations are more stable in the group closer to physiological concentrations (INS0.1), a general inactivation of insulin does not seem plausible.

However, different reaction and activity profiles in vivo and in vitro are plausible and need to be considered, as in vitro models often aim to investigate consequences with new methods and expensive equipment where an insufficient concentration of the tested factor could cause problems to evaluate the data. More studies focusing on the molecular response of embryos to insulin should be aimed to obtain a better understanding of exposure to insulin during early embryonic development. Unnecessary high doses of insulin should be avoided as the biological consequences of an uncritical use of supra-physiological doses are not clearly known.

## Abbreviations

AM: after maturation
AME: after maturation empty
BC: blastocyst
BM: before maturation
BSA: bovine serum albumin
COC: cumulus oocyte complex
DELFIA: dissociation-enhanced lanthanide fluoro immunoassay
ED50: median effective dose
EIA: enzyme immunoassay
ELISA: enzyme-linked immunosorbent assay
FSH: follicle-stimulating hormone
ITS: insulin/transferrin/sodium selenite
IVM: in vitro maturation
IVP: in vitro production
LH: luteinizing hormone
RIA: radio immunoassay
SOF: synthetic oviductal fluid
